# A Survey of the Gene Repertoire of *Gigaspora rosea* Unravels Conserved Features among Glomeromycota for Obligate Biotrophy

**DOI:** 10.3389/fmicb.2016.00233

**Published:** 2016-03-01

**Authors:** Nianwu Tang, Hélène San Clemente, Sébastien Roy, Guillaume Bécard, Bin Zhao, Christophe Roux

**Affiliations:** ^1^State Key Laboratory of Agricultural Microbiology, College of Life Science and Technology, Huazhong Agricultural UniversityWuhan, China; ^2^CNRS, Laboratoire de Recherche en Sciences Végétales, UMR, Université Paul Sabatier – Université de ToulouseCastanet Tolosan, France; ^3^AGRONUTRITION Laboratoire de BiotechnologiesToulouse, France

**Keywords:** arbuscular mycorrhizal fungi, Glomeromycota, obligate biotrophy, gene repertoire, mRNA sequencing, phylogenomics

## Abstract

Arbuscular mycorrhizal (AM) fungi are a diverse group of soil fungi (Glomeromycota) that form the most ancient mutualistic association termed AM symbiosis with a majority of land plants, improving their nutrition uptake and resistance to stresses. In contrast to their great ecological implications, the knowledge of the molecular biological mechanisms involved is still scant, partly due to the limited genomic resources available. Here, we describe the gene repertoire of a new AM fungus *Gigaspora rosea* (Diversisporales). Among the 86332 non-redundant virtual transcripts assembled, 15346 presented similarities with proteins in the Refseq database and 10175 were assigned with GO terms. KOG and Interpro domain annotations clearly showed an enrichment of genes involved in signal transduction in *G. rosea*. KEGG pathway analysis indicates that most primary metabolic processes are active in *G. rosea*. However, as for *Rhizophagus irregularis*, several metabolic genes were not found, including the fatty acid synthase (FAS) gene. This finding supports the hypothesis that AM fungi depend on the lipids produced by their hosts. Furthermore, the presence of a large number of transporters and 100s of secreted proteins, together with the reduced number of plant cell wall degrading enzymes could be interpreted as an evolutionary adaptation to its mutualistic obligate biotrophy. The detection of meiosis-related genes suggests that *G. rosea* might use a cryptic sexual process. Lastly, a phylogeny of basal fungi clearly shows Glomeromycota as a sister clade to Mucoromycotina, not only to the Mucorales or Mortierellales. The characterization of the gene repertoire from an AM fungal species belonging to the order of Diversisporales and its comparison with the gene sets of *R. irregularis* (Glomerales) and *Gigaspora margarita* (Diversisporales), reveal that AM fungi share several features linked to mutualistic obligate biotrophy. This work contributes to lay the foundation for forthcoming studies into the genomics of Diversisporales, and also illuminates the utility of comparing gene repertoires of species from Diversisporales and other clades of Glomeromycota to gain more insights into the genetics and evolution of this fungal group.

## Introduction

Arbuscular mycorrhizal (AM) fungi, belonging to the phylum Glomeromycota, participate in the most widespread mutualistic beneficial association, the so called AM symbiosis, with a majority of land plants including most major crops, thus making themselves of great incidence and interest in natural and cultivated ecosystems (Parniske, 2008; Sawers et al., 2008). As obligate biotrophs, AM fungi depend on host-derived carbon compounds to complete their biological cycle: it was estimated that up to 20% of plant photosynthates are transferred to AM fungi (Jakobsen and Rosendahl, 1990). In return for this high carbon cost, AM fungi significantly improve plant nutrition with enhanced water and soil nutrient uptake: up to 90% of phosphorus and nitrogen plant requirements are supplied by AM fungi (Smith and Read, 2008). This reciprocal reward mechanism is assumed to ensure a “fair trade” between the symbiotic partners (Kiers et al., 2011; Fellbaum et al., 2012). Probably due to this reciprocal reward mechanism, AM symbiosis has been stabilized for more than 400 million years, thereby is thought to be the most ancient plant symbiosis in nature (Remy et al., 1994; Humphreys et al., 2010).

Establishing a functional AM symbiosis entails sequential modifications in both AM fungus and plant partners, at both physiological and molecular levels (Bonfante and Genre, 2010). Using forward and reverse genetic approaches light is beginning to be shed on the plant molecular events during the development of AM symbiosis (Harrison, 2012; Gutjahr and Parniske, 2013), but the fungal molecular bases that underlie this process remain largely unknown owing to the complexity of their biology and genetics (Lanfranco and Young, 2012). The obligate biotrophy of AM fungi strongly limits their suitability for molecular studies, and the mechanisms that maintain these intracellular symbionts as obligate biotrophs remains to be elucidated. Due to the lack of observed sexual structures, AM fungi are generally assumed to be asexual organisms. However, the identification in some species of several genes usually involved in fungal meiosis hints at the existence of a cryptic sexual cycle (Halary et al., 2011; Tisserant et al., 2012, 2013). AM fungi are aseptate and coenocytic, and high genetic variation of ribosomal and protein-coding genes had been reported within individual spores. Nevertheless, whether the genetic variations happen within each nucleus or between nuclei (i.e., homokaryosis vs. heterokaryosis) is still in a controversy (Kuhn et al., 2001; Pawlowska and Taylor, 2004; Hijri and Sanders, 2005; Pawlowska, 2005; Stukenbrock and Rosendahl, 2005; Bever et al., 2008; Sanders and Croll, 2010; Boon et al., 2015).

Thanks to the advance in sequencing technology, the first transcriptome and genome of an AM fungus *Rhizophagus irregularis* DAOM197198 were released in recent years (Tisserant et al., 2012, 2013; Lin et al., 2014). These genome-wide studies revealed far more information than ever before about the AM fungal gene repertoire and expression dynamics across its life cycle, such as the reduced number of genes involved in plant cell wall degradation, over-represented genes involved in signaling pathway and highly induced transporter and small secreted peptide genes during symbiosis. Moreover, these datasets also started to shed light on the key issues mentioned above. For instance, the low level of polymorphism detected in the genome of *R. irregularis* DAOM197198 clearly favored the homokaryotic hypothesis (Tisserant et al., 2013; Lin et al., 2014). Besides, the absence of several metabolic genes suggests a strong dependency on certain metabolites or precursors delivered by the host plant, a feature that may explain the obligatory biotrophism. These recent results underline the effectiveness of AM fungal genomics in providing clues to decipher the biological mechanisms of AM symbiosis at the molecular level. However, it must be pointed out that all this evidence only comes from a single strain of one AM fungal species belonging to one of the four Glomeromycota orders. Given the phylogenetic diversity observed in Glomeromycota (Schüβler et al., 2001; Krüger et al., 2012), the features identified in *R. irregularis* might be extremely specific and not reflect the biology of the AM fungi as a group. To determine the conservation of these features and broaden our knowledge on AM fungi and AM symbiosis, it is now critical to survey other AM fungal species belonging to divergent phylogenetic clades in Glomeromycota.

Because of the large individual size and multi-geotropism of germinating hyphae, *Gigaspora* sp. (belonging to Diversisporales, Gigasporaceae) have long been used as ideal models to study the responses to plant chemical signals (Buee et al., 2000; Akiyama et al., 2005; Besserer et al., 2006). Moreover, the life history and functional traits of Gigasporales species appear to be different from those of the Glomerales species (including *R. irregularis*). For example, while the Glomeraceae species colonize host roots rapidly and invest more biomass inside the host root, the Gigasporaceae species have a relatively slower colonization rate and higher ratio of extraradical hyphae to intraradical hyphae (Parniske, 2008). This difference could explain the higher scavenging ability for soil nutrients of Gigasporaceae (Maherali and Klironomos, 2007). Probably due to these traits, the Gigasporaceae species might be largely unexplored with current sampling and research methods which has a severe bias toward the species colonizing rapidly and investing more biomass inside the root (Öpik et al., 2010). For these reasons, *Gigaspora* sp. represent AM fungal species of great interest to be investigated, in order to broaden our knowledge of AM fungi at the physiological, ecological, and genetic level. In line with this, the transcriptome of *Gigaspora margarita* was assembled very recently (Salvioli et al., 2016). In order to strengthen the identification of shared and AM fungal lineage specific features, we surveyed the gene repertoire of *Gigaspora rosea* DAOM194757. mRNAs were isolated from several biological conditions (germinating spores, symbiotic tissues, extraradical hyphae) and sequenced using the Illumina strategy. The transcribed gene set of *G. rosea* was assembled from more than 230 M sequencing reads (over 24 Gbs). We then detailed the gene repertoire of *G. rosea*, with a focus on the gene categories that could be related to the specific biology of AM fungi. Finally, using the data retrieved from this gene set, a phylogeny based on a multiple-gene alignment was inferred to clarify the phylogenetic placement of Glomeromycota within the basal fungal lineages.

## Materials and Methods

### Preparation of Biological Materials

To ensure a gene repertoire as complete as possible, biological materials were prepared in a manner to cover several typical fungal developmental stages: germinating spores with or without the addition of root exudates or GR24, a synthetic analog of strigolactones that were characterized as plant signals perceived by AM fungi (Akiyama et al., 2005; Besserer et al., 2008), extraradical mycelium and symbiotic root tissues (detailed information available at NCBI GEO portal, [GEO: GSE67906] and Supplementary Table S1). In brief, spores and extraradical mycelium of *G. rosea* DAOM 194757 produced on root organ cultures were purchased from Agronutrition (Carbone, France). For the germinating spores treated with GR24, batches of 500 spores were incubated for pre-germination during 5 days in liquid mineral M medium (Bécard and Fortin, 1988) under 30°C, 2% CO_2_ with or without (control) GR24 at the final concentration of 10^-6^ M during 2 days before sample collection. Although, GR24 induces *G. rosea* branching at concentration as lower as 10^-13^ M (Besserer et al., 2006), assays were performed at 10^-6^ M due to GR24 lability in water even at acidic pH (half-life time of 9 h at pH 5.9, Rasmussen et al., 2013). More, stimulatory activity of GR24 on *G. rosea* increases with concentration and no inhibitory effect on AM fungal growth was observed with strigolactones at concentration up to 10^-5^ M (Besserer et al., 2006). For the root exudates experiment, batches of 500 spores were co-cultivated with *in vitro* cultured carrot roots but separated with a cellophane membrane, for 10 days at 30°C and 2% CO_2_. Symbiotic roots with intraradical hyphae (the presence of some running extraradical hyphae attached to root surface could not be excluded) were collected from 4 to 6 weeks-old mycorrhizal *Medicago truncatula* (A17) and *Brachypodium distachyon* (Bd21; growth conditions: 25°C, 16 h of day and 22°C, 8 h of night).

### *Gigaspora rosea* mRNA Sequencing and Assembly

*Gigaspora rosea* RNA was extracted from the different biological conditions using RNeasy Plant Mini RNA Extraction Kit (Qiagen, Germany). Library construction and sequencing were performed on the GeT platform (^1^Toulouse, France) according to the standard Illumina protocols. Totally, 31 libraries were constructed for all the samples mentioned above (see Supplementary Table S1). Short pair-end sequencing reads generated from illumina platforms (2 × 101 bp from Hiseq2000 and 2 × 151 bp from Miseq1000) were trimmed based on the quality scores (limit: 0.05), end ambiguity (maximum allowed number of ambiguities: 2) and adaptor sequences, using the commercial software CLC Genomic Workbench (v6.0, Denmark). The reads less than 50 bp after trimming were discarded. In addition, as the symbiotic materials contained RNAs from the host plant and other possible contaminants (fungi and other eukaryotic microorganisms), we developed a strategy to remove these non-*G. rosea* reads before assembly (Supplementary Figure S1A). The cleaned symbiotic reads displayed a GC% distribution highly similar to that of the non-symbiotic pure fungal reads (Supplementary Figure S1B), validating the efficiency of our procedure. We used these cleaned symbiotic reads and non-symbiotic fungal reads for the assembly process, also using the CLC software with default settings. In order to improve the quality of these assembled contigs, the option of mapping reads back onto the assembled contigs was run with default settings. See the online CLC protocol for more details on the reads trimming and assembly^2^. The raw sequencing reads, assembly and annotations have been deposited in NCBI-GEO database (GEO: GSE67906 and GSE67911).

### SNP and SSR Identification

To detect the single nucleotide polymorphisms (SNPs) in the transcripts, the trimmed reads in pairs from all samples were firstly mapped onto all the transcripts (length fraction: 0.9 and similarity: 0.95). Based on the mapping, the procedure of quality-based variants detection in CLC genomics workbench software was run using default quality settings and advanced significance settings of required variant count 3; sufficient variant count 5. Only SNPs with a frequency of not less than 0.02, forward/reverse balance greater than 0.05 and average base quality more than 20 were called. Whilst, the simple sequence repeats (SSRs) of 2–6 nucleotides and total length ≥20 bp, that is a minimum of 10 dimers, 7 trimmers, 5 tetramers, 4 pentamers or 4 hexamers, were identified using the SSR Locator tool (Costa De Oliveira et al., 2008).

### Functional Annotation

Prior to the annotation process, the representativeness of *G. rosea* transcriptome assembly was both assessed by CEGMA and BUSCO methods (Parra et al., 2009; Simão et al., 2015). A total of 248 core eukaryotic genes were searched against *G. rosea* assembly as described in Tisserant et al. (2012). A total of 429 groups of eukaryotic BUSCO genes and 1438 groups of fungal BUSCO genes were searched against *G. rosea* assembly using the mode of *trans* with default settings. Annotations were derived by comparing the transcripts to the public Refseq protein database with the blastx algorithm (*e*-value cutoff at 1E^-10^). All protein ORFs (including the complete and incomplete ones) encoded by *G. rosea* were predicted by the OrfPredictor tool using default settings (Min et al., 2005). The Gene ontology (GO) terms were assigned to each transcripts using InterProScan program (Quevillon et al., 2005). The annotation of Eukaryotic Orthologous Groups (KOG) and function classes was derived using webMGA tool (Wu et al., 2011). Potential InterPro domains harbored in each transcript were predicted based on InterProScan hits. *G. rosea* metabolic pathway prediction was achieved by the online KEGG Automatic Annotation Server (KAAS) using the single-directional best-hit information method and default bitscore threshold (Moriya et al., 2007). The following organisms: uma, spo, tml, pcs, ang, ani, bfu, ncr, mgr, fgr, sce, yli, ecu, mgl, mpr, ppl, cne, ure, cpw, aor, afm, ssl, ago, kla, ppa, vpo, cgr, and dha were selected as the reference datasets. Carbohydrate-active enzymes (CAzymes) in the predicted proteome were annotated by the dbCAN annotation server, with an *e*-value cutoff at 1E^-10^ (Yin et al., 2012). To predict the secretome of *G. rosea*, the SignalP 4.1 server^3^ was used to check the presence of signal peptide and then the TMHMM 2.0 online server^4^ was adopted to predict the presence of transmembrane helices in all *G. rosea* proteins. Both processes were run with the default settings and score cutoff (D-score > 0.450). The proteins containing a signal peptide but without any transmembrane domains (parameter PredHel = 0) were further analyzed using TargetP 1.1 tool (Emanuelsson et al., 2007) and only the proteins strongly predicted to be located in the secretory pathway (parameter LOC = S and Reliability class = 1) were considered as the secreted proteins (SPs). Only proteins longer than 30 amino acids and starting with a methionine were included in this secretome prediction. Membrane transporters were identified from the *G. rosea* gene repertoire by blastx searches against the Transporter Classification Database^5^ with an *e*-value cut-off at 1E^-20^, as described previously (Wang et al., 2015). The *G. rosea* transcripts coding for the meiosis required genes were obtained by tblastn against the whole gene repertoire using the inventory of 86 genes reported in the study of Halary et al. (2011), as the queries.

### Phylogenomic Analysis

A fungal phylogeny was inferred from a set of orthologous genes among 35 fungal taxa from most basal lineages. These species are: *R. irregularis* DAOM197198 (Tisserant et al., 2013) and *G. margarita* BEG34 (Salvioli et al., 2016) of Glomeromycota; *Mortierella elongata*, *Mortierella verticillata*, *Phycomyces blakesleeanus* NRRL1555, *Rhizopus oryzae* 99-880 (Ma et al., 2009) and *Mucor circinelloides* CBS277.49 of Mucoromycotina; *Saccharomyces cerevisiae* S288C (Goffeau et al., 1996), *Candida albicans* SC5314 (Jones et al., 2004; Braun et al., 2005), *Aspergillus nidulans* (Galagan et al., 2005; Arnaud et al., 2012), *Magnaporthe grisea* strain 70-15 (MG8; Dean et al., 2005), *Neurospora crassa* OR74A (Galagan et al., 2003), *Botrytis cinerea* strain B05.10 (Amselem et al., 2011; Staats and van Kan, 2012), *Blumeria graminis* f.sp.hordei strain DH14 (Spanu et al., 2010), *Tuber melanosporum* (Martin et al., 2010) and *Schizosaccharomyces pombe* (Wood et al., 2002, 2012) of Ascomycota; *Coprinopsis cinerea* strain Okayama 7 (#130; Stajich et al., 2010), *Laccaria bicolor* S238N-H82 (Martin et al., 2008), *Cryptococcus neoformans* var.grubii (Janbon et al., 2014), *Piriformospora indica* DSM 11827 (Zuccaro et al., 2011), *Ustilago maydis* (Kämper et al., 2006), *Puccinia graminis* and *Melampsora larici-populina* (Duplessis et al., 2011) of Basidiomycota; *Spizellomyces punctatus* DAOM BR117 and *Batrachochytrium dendrobatidis* JAM81 of Chytridiomycota; *Encephalitozoon cuniculi* GB-M1, *Nosema ceranae* BRL01, *Enterocytozoon bieneusi* H348, *Antonospora locustae* HM-2013 and *Nematocida parisii* ERTM1 of Microsporidia; *Rozella allomycis* CSF5 of Cryptomycota. The four Holozoa species *Amphimedon queenslandica*, *Nematostella vectensis*, *Monosiga brevicollis* and *Capsaspora owczarzaki* were used as outgroups. Their proteomes were retrieved either from JGI Genome Portal (Nordberg et al., 2014), Broad Institute of Harvard and MIT^6^ or from the SGD^7^. The 118 orthologous gene groups used for the phylogomics were retrieved using the SPOCS pipeline with default settings (Curtis et al., 2013). Multiple sequence alignments were built for each gene using the MAFFT algorithm (Katoh et al., 2002) and then concatenated into a super matrix of 94153 sites in MEGA6 (Tamura et al., 2013). After removing poorly aligned positions and divergent regions in the super matrix using Gblocks-0.91b (Castresana, 2000) with the relaxed parameters, the resulting alignment of 35313 amino acid sites was used to infer the phylogenetic trees. The phylogeny was inferred by using both Bayesian (MrBayes, v3.2.3) method (Ronquist and Huelsenbeck, 2003) and Maximum-Likelihood (ML) method PhyML 3.0 (Guindon et al., 2010). The best amino acid substitution model MTrev+G4, determined by TOPALi software (Milne et al., 2009), was chosen for both inferences. Bayesian Metropolis coupled Markov chain Monte Carlo analyses (B-MCMCMC) consisted of two independent runs and four chains to ensure it remained stationary and convergent toward the same log-likelihood level. We sampled one of 50 trees during 100 000 generations and the last 1500 trees sampled from each run (burnin = 500) were used to build the majority-rule consensus tree. Branch support was considered as significant only if the posterior probabilities (PPs) were not less than 0.95. Phylogeny inference based on the ML method was performed with 100 bootstraps on the Phyml server^8^. The phylogenetic tree was displayed with the Figtree program (v1.4.2).

## Results and Discussion

### *Gigaspora rosea* DAOM 194757 Gene Repertoire Generation

*De novo* assembly of 230 084 705 reads (208 454 640 non-symbiotic pure fungal reads plus 21 630 065 cleaned symbiotic fungal reads, see Materials and Methods) produced 97463 contigs with an average length of 611 bp (N_50_: 839 bp, 33% of GC content, total length: 59.5 Mb) and more than 90% of these reads could be mapped back to the assembly. Since symbiotic tissues were obtained from pot cultures, this original assembly still contained some non-*G. rosea* contaminant contigs. We compared the 97463 contigs with the NCBI nr/nt DNA database and removed 11131 sequences showing high similarity (blastn using a stringent *e*-value cut-off at 1E^-50^, as used by Tisserant et al., 2012) to those from organisms other than fungi: 7328 Eukaryots (most abundant *Acanthamoeba castellanii*, 449 NRVTs), 2819 bacteria (most abundant *Bacteroides finegoldii*, 613 NRVTs), 27 archaebacteria and 111 virus (most abundant *Megavirus iba*, 51 NRVT). The remaining 86332 contigs constitute the first transcribed gene set of *G. rosea* (GiroV1, Girov1.fa available at the NCBI GEO portal [GEO: GSE67906]). The *G. rosea* gene repertoire GiroV1 is approximately 55.5 Mb in size, with an average contig length of 643 bp (N_50_: 948 bp and 33% of GC content). CD-HIT clustering analysis indicated that *G. rosea* contigs and the predicted proteins are not redundant at the sequence level (identity threshold: 0.95, Li and Godzik, 2006), although it cannot be excluded that redundancy can be masked by incomplete assembling (non-recovering contigs from the same full-length gene). In absence of size cut-off, the number of contigs in *G. rosea* is in the same range than the recent sequenced *G. margarita* (Salvioli et al., 2016). While considering the sequences longer (≥) than 350 bp, the NRVTs and gene repertoire of *G. rosea*, *G. margarita* and *R irregularis* are respectively composed by 46,112, 86,183, and 22,069 contigs (at the threshold of 1000 bp, the numbers are respectively 13,327, 34,011, and 8,232). Although, the number of defined gene models in *Gigaspora* sp. is still hypothetical due to the lack of corresponding genome assembly, they both have a transcribed gene set greater than that of *R. irregularis.* A larger genome size has been estimated in *Gigaspora* sp. (ca. 700 Mb; Hosny et al., 1998) as compared to *R. irregularis* (ca. 150 Mb; Sęedzielewska et al., 2011; Tisserant et al., 2013; Lin et al., 2014). Although, it was already reported in fungi that genome expansion is not proportionally correlated to gene repertoire increase (Kelkar and Ochman, 2012), it can be hypothesized that genome inflation in *Gigaspora* sp. has been accompanied to some extent by gene duplication and/or gene family expansion.

The representativeness of the GiroV1 assembly was assessed in two ways. First, we found that among 428 *G. rosea* ESTs downloaded from the NCBI database, 307 can be blasted onto the GiroV1 contigs (*e*-value cut-off at 1E^-50^ as used by Tisserant et al., 2012). The 121 ESTs that do not match in GiroV1 are mainly short sequences. Secondly, 240 of the 248 Core Eukaryotic Genes (CEGs, *e*-value cut-off at 1E-5; Parra et al., 2009), and 378 of the 429 BUSCO genes (Simão et al., 2015) were found to be present in GiroV1, suggesting this assembly covers most of the genes of *G. rosea* (Supplementary Table S2A). This was further supported by the finding that most of the KEGG pathways identified in the genome of *R. irregularis* and *G. margarita* were covered by GiroV1 assembly (Supplementary Table S2B). Therefore, these 86,332 contigs were considered to be the non-redundant virtual transcripts (NRVTs) that will be used for further SNP and SSR analyses and functional annotations.

### Simple Sequence Repeat (SSR) Loci Identification

Simple sequence repeat also known as microsatellite, as a versatile molecular marker has been used in several population genetics studies of AM fungi (Longato and Bonfante, 1997; Croll et al., 2008; Mathimaran et al., 2008). We identified in GiroV1 1071 SSR loci (≥20 bp) distributed in 994 NRVTs, including 236 dimers, 393 trimers, 213 tetramers, 127 pentamers, and 102 hexamers. As observed in the whole gene set, the A/T-rich motifs are the most abundant among the identified SSRs (Supplementary Table S3). To date, although most population genetics studies of AM fungi have focused on the *Glomus*/*Rhizophagus* species probably because of their prevalence in the sampled sites and sampling bias (Öpik et al., 2010), the SSR loci identified here would provide a foundation for developing useful genetic markers in the future when more *Gigaspora* species and strains are collected from root and soil.

### Single Nucleotide Polymorphism (SNP) Analysis Suggests a Homokaryotic Organization of *G. rosea* Nuclei

**FIGURE 1:**
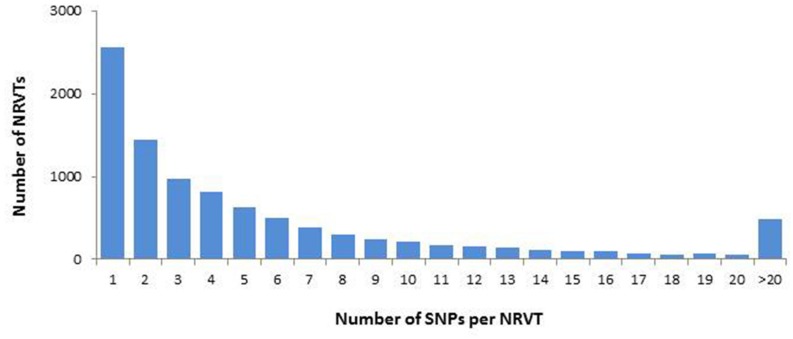
**Single nucleotide polymorphism (SNP) distribution in GiroV1 assembly.** SNPs were identified by the quality-based variant detection procedure in CLC software and the number of NRVTs containing various densities of SNPs were calculated in GiroV1.

During their life cycle, AM fungi form multinucleate spores and no mono-nuclear stage was observed. These propagative structures are thought to be asexual spores where high genetic variations occur between co-existing nuclei (Sanders and Croll, 2010). However, the first available genomic data did not support this interpretation (Tisserant et al., 2013; Lin et al., 2014). To check it on *G. rosea*, we examined genetic diversity in the gene set of *G. rosea* through SNP analysis. The quality-based variant detection procedure in CLC software called a total of 57405 SNPs distributed in 9576 NRVTs (11.1% of the total NRVTs), with an average of 1 SNP per kb. Few NRVTs are highly polymorphic: only 1499 of the 9576 NRVTs contained >10 SNP loci (**Figure 1**). The detection of multiple SNP loci in 1000s of *G. rosea* NRVTs confirmed the previous finding that genetic variation exists within AM fungus individuals, at not only the genome level but also the transcriptome level (Boon et al., 2010, 2015; Tisserant et al., 2012, 2013). In the absence of genomic assembly, the SNP origins remain undefined, i.e., possibly either from different copies of genes on a single haploid nucleotype, from divergent alleles on a single polyploid nucleotype or from single-copy genes on different nucleotypes. Considering: (i) the large genome size of Gigasporaceae (Hosny et al., 1998) that could lead to gene family expansion, (ii) the fact that 96.3% of NRVTs contain no or only less than 5 SNPs, and (iii) the lower level of SNPs compared to true heterokaryotic fungi (>10 SNPs per kb; Hane et al., 2014), it can be suggested that the nuclear organization of *G. rosea* DAOM194757 is mostly homokaryotic as observed in *R. irregularis* DAOM197198.

### Global Annotation

The homology search against the Refseq protein database resulted in only 15,346 annotated *G. rosea* NRVTs (17.8% of the total 86,332 NRVTs) at *e*-value cut-off of 1E^-10^ (file Girov1_annotation.xlsx.gz available at GEO portal [GEO: GSE67906]). Using same threshold, 33.7% of *R. irregularis* NRVT had a hit on GenBank nr (Tisserant et al., 2012). That such a small portion of *G. rosea* NRVTs could be annotated by the already known proteins in other organisms is not unexpected, since a large number of genes have been shown to be AM lineage specific (Tisserant et al., 2012, 2013; Lin et al., 2014; Salvioli et al., 2016) and there are only limited AM fungal sequences in public databases. We observed that the number of matched NRVTs increased (22393, at the same *e*-value cut-off) when searching against the proteins of *R. irregularis* DAOM197198, the only AM fungal genome currently available. On the other hand, as 74% of the NRVTs did not match between the gene repertoires of these two species belonging to Glomeromycota, it indicates that most of the genes are lineage-specific, underlining the necessity to investigate alternative species to *R. irregularis*. We found 54,883 NRVTs of *G. rosea* NRVTs (64%, tblastx with the *e*-value < 1E^-10^) that may be homologous to the transcripts of *G. margarita*, since they belong to the same genus.

**FIGURE 2:**
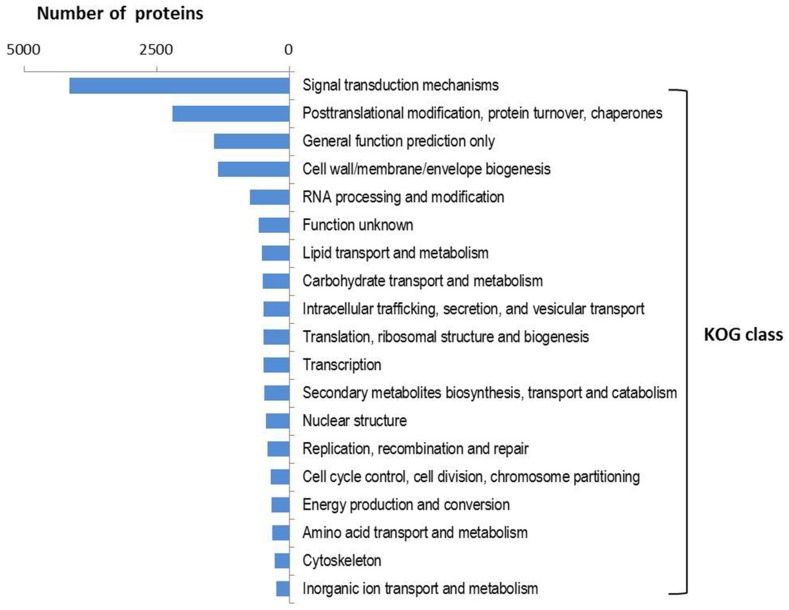
**KOG function class distribution in the predicted proteome of *G*. *rosea*.** A total of 2961 KOG groups were assigned to 11505 *G. rosea* proteins using the webMGA tool with an *e*-value cut-off of 1E^-5^. The distribution of 25 KOG function classes to which these KOG groups belong is summarized as the percentages.

InterProscan results assigned GO terms (including biological process, molecular function, and molecular component) to 10175 NRVTs. Of these terms, the metabolic process and cellular process were the most represented biological processes whereas binding and catalytic activity represented the most abundant molecular functions (Supplementary Figure S2). Among the 86332 NRVTs, open reading frames (ORFs, complete or incomplete) were predicted for 83360 NRVTs by the OrfPredictor tool, and these proteins were classified into 25 KOG functional classes in which the class of signal transduction mechanisms was strikingly overrepresented (accounting for 36.0% of total annotated proteins, **Figure 2**). Consistently, Interpro domains (for instance, IPR001245: serine-threonine/tyrosine-protein kinase catalytic domain and IPR000719: protein kinase, catalytic domain) involved in signaling were significantly enriched in *G. rosea* proteins (Supplementary Figure S3). Noticeably, the significant enrichment of proteins involved in signaling is also the case in *R. irregularis* and *G. margarita* (Tisserant et al., 2013; Lin et al., 2014; Salvioli et al., 2016; Supplementary Table S4), even of the ectomycorrhizal fungus *Laccaria bicolor* (Martin et al., 2008) thus indicating the involvement of intensive signaling processes as adaptive mechanisms to mycorrhizal symbiosis. Besides, our KOG analysis found several protein families with different abundances in *G. rosea*, *G. margarita* and *R. irregularis* (Supplementary Table S5). For instance, the Ran GTPase-activating protein family (KOG1909) was significantly enriched in the two *Gigaspora* species when compared with *R. irregularis*. Ran GTPase-activating protein (RanGAP) is a GTPase activator for the nuclear Ras-related regulatory protein Ran, converting it from the active GTP-bound state to the inactive GDP-bound state. Ran is an abundant GTPase that is highly conserved in eukaryotic cells and has been implicated in many aspects of nuclear structure and function during the eukaryotic cell division cycle (Clarke and Zhang, 2001). Although the exact functions played by these RanGAPs and other proteins are not known in AM fungi, the difference in protein family size involved in signaling pathways according to AM fungal species might be one of the mechanisms contributing to their specific adaptive traits.

### Missing Glomeromycota Core Genes (MGCCs) in Girov1: Causes of Obligate Biotrophy?

KEGG pathway analysis assigned a total of 8397 NRVTs into 3010 KEGG orthology (KO) groups that cover most of the primary metabolism enzymes on the map (see Supplementary Table S2), suggesting that most primary metabolic processes are present in *G. rosea*. However, some major genes involved in primary metabolism seem to be absent from GiroV1. In Supplementary Table S6, comparing the missing gene list of *G. rosea* to those of *G. margarita* and *R. irregularis* (Tisserant et al., 2012, 2013; Salvioli et al., 2016) suggests a strong conservation of the mechanisms underlying obligate biotrophy among AM fungi. This observation leads to a suggested Missing Glomeromycota Core genes (MGCGs) as proposed for ascomycetes with the missing ascomycete core genes (MACGs) by Spanu et al. (2010). The most striking feature among MGCGs is the absence of the type I FAS gene, as pointed out for *R. irregularis* (Wewer et al., 2014). It was previously demonstrated that *G. rosea* was unable to synthetize palmitic acid in the absence of a host, although labeled carbon assays indirectly suggested that the fungus synthesizes its own palmitate in symbiotic tissues (Pfeffer et al., 1999; Trépanier et al., 2005). As an oleic fungus full of lipids, the absence of type I FAS suggests that lipids are derived from plant metabolism, particularly from the plastids surrounding the arbuscules in host cells (Strack and Fester, 2006).

The MGCGs list also includes the invertase activity, involved in the cleavage of sucrose, which is the major sugar form produced during photosynthesis and translocated to the roots, into fructose and glucose (Tauzin and Giardina, 2014). GiroV1 presents several putative hexose transporters where the importance of monosaccharide transport in the establishment of AM symbiosis has already been demonstrated (Helber et al., 2011). The absence of an invertase gene suggests that *G. rosea* is dependent on its host for sucrose production and hydrolysis, consistently with host invertase induction in periarbuscular spaces (Schaarschmidt et al., 2006). It might seem surprising for a fungus that needs glucose as a carbon source to be dependent on host invertase activity for sucrose hydrolysis. It was previously reported that most pathogenic fungi possess invertase genes whereas ectomycorhizal fungi usually don’t (Parrent et al., 2009). Moreover, recent evidence points out that the sucrose/glucose ratio and host cell wall invertase activity play an essential role in triggering the plant defense responses (Tauzin and Giardina, 2014). Additionally, Schaarschmidt et al. (2006, 2007a,b) found that only mild upregulation of plant invertase gene expression was induced upon mycorrhization. Based on these results, it is then tempting to speculate that the absence of fungal invertase and the fine regulation of plant invertase activity by AM fungi could be an evolutionary adaptation to lowering host immunity and thus maintaining a “long-term” interaction (Tauzin and Giardina, 2014).

Several NRVTs among MGCGs are involved in thiamine biosynthesis. Thiamine is a cofactor involved in decarboxylation steps required in several metabolic pathways such as the catabolism of sugars and amino acids (Kohlmeier, 2003; Goyer, 2010). In addition, various genes involved in uracil transport, detoxification or stress responses, ER (endo-reticulum) quality control and aromatic amino acid metabolism were also missing in *G. rosea*. Loss of some genes is typically a feature shared by several plant and human obligate biotrophic pathogens (Spanu et al., 2010; Duplessis et al., 2011; Cisse et al., 2014). This strongly suggests that the deletion of these genes might be one of the reasons for the obligate biotrophy of these organisms. However, unlike these obligate biotrophic pathogens, *G. rosea* has retained the genes involved in nitrate metabolism and sulfate assimilation. These features, together with the rare presence of genes matched to the secondary metabolism pathways, agree with the finding in the genome of *R. irregularis* (Tisserant et al., 2013).

### Carbohydrate-Acting Enzymes (CAZymes) Annotation

A reduced pool of carbohydrate-acting enzymes (CAZymes) involved in the degradation of plant cell wall polysaccharides has been reported in several obligate biotrophic pathogens and mutualists including ectomycorrhizal and AM fungi (Martin et al., 2008, 2010; Spanu et al., 2010; Duplessis et al., 2011; Tisserant et al., 2012, 2013; Kohler et al., 2015; Salvioli et al., 2016). Through dbCAN HMMER search, we found 293 *G. rosea* NRVTs that encode a total of 64 CAZyme families including 26 glycosyltransferases (GTs), 20 glycoside hydrolases (GHs), 6 carbohydrate esterases (CEs), 6 carbohydrate-binding modules (CBMs), and 6 auxiliary activities (AAs; Supplementary Table S7). Consistent with the aforementioned lack of an invertase gene in *G. rosea*, the whole sucrose hydrolase family GH32 was also absent. Polysaccharide lyases (PLs) involved in the degradation of pectin, and lytic polysaccharide monooxygenases (LPMOs) AA9 (formerly GH61), AA10 (formerly CBM33) were absent. Except for GH5 and GH9, no other cellulose degrading enzymes like cellobiohydrolase (GH6, 7) and β-1,4-glucosidase (GH1, 3) genes were detected in *G. rosea* transcriptome. Similarly, most GH families acting on hemicellulose (GH2, 3, 10, 11, 12, 26, 43, 74) and pectin (GH2, 3, 28, 43, 51, 53, 54, 78, 88, 105) were also missing. This reduced profile of CAZymes encoded by *G. rosea* is very similar to that in the genome of *R. irregularis* (Tisserant et al., 2013). Such a reduced set of CAZymes encoded by AM fungi could be an evolutionary adaptation to the symbiotic lifestyle, to avoid the detection of PAMPs (pathogen-associated molecular patterns) or DAMPs (damage-associated molecular pattern molecules) by the host immune system through the minimum release of fragments resulting from polysaccharide degradation (Tisserant et al., 2012, 2013; Malinovsky et al., 2014).

### Secretome Prediction

Pathogens of all classes including viruses, bacteria, fungi, and oomycetes present SPs (SPs), in their genome, some of them acting as effectors by interfering with host pathways, mainly immune-related, to facilitate successful infection (Guttman et al., 2014). The recent finding of many SPs, among which few have been validated as effectors, in ectomycorrhizal and AM fungi suggests that symbionts might also have employed a similar vocabulary, as pathogens to dialog with their hosts (Martin et al., 2008; Kloppholz et al., 2011; Tisserant et al., 2013; Lin et al., 2014). In our analysis, among the total encoded 83360 ORFs predicted in GiroV1, we identified 441 potential SPs (Supplementary Table S8). This number is comparable with that in the genome of *R. irregularis* which was predicted to contain up to 566 SPs, using similar tools and thresholds (Tisserant et al., 2013; Lin et al., 2014). Function could be inferred for 141 *G. rosea* SPs through homology searches against Refseq, Swissprot and Fungal Secretome Knowledgebase (FunSecKB2) databases (Meinken et al., 2014). Interestingly, we observed that the secretome of *G. rosea* overlapped the most with that of the Mucoromycotina fungus *Rhizopus delemar* in the FunSecKB2 database (14 *G. rosea* SPs showing similarity with *Rhizopus* SPs, then five SPs with *Melampsora larici-populina* ones, see Supplementary Table S8), thus hinting at a possible close relationship between them. GO analysis showed that the *G. rosea* SPs are involved in diverse biological processes such as proteolysis, protein maturation, carbohydrate metabolic process, and oxidation–reduction. CAZyme annotation suggests that the carbohydrate metabolic process related SPs could be involved in the degradation of bacterial cell wall peptidoglycan (like the lysozyme family GH25) and plant cell wall polysaccharides (like the α-galactosidase family GH27). Comparison of secretomes between *G. rosea* and *R. irregularis* identified 121 *G. rosea* SPs that have at least one homolog in *R. irregularis* (blastp, *e*-value < 1E^-5^). Among these common SPs, thirty might also be involved in pathogen-host interactions (blastp against PHI-base, with *e*-value < 1E^-5^; Winnenburg et al., 2006), indicating shared roles with various fungus-plant associations. Lin et al. (2014) identified a tribe of 22 AM fungus-specific SPs that were only present in *R. irregularis* but not in pathogens. In our analysis, four of these 22 SPs were also found in the *G. rosea* secretome, suggesting their important and conservative roles in AM symbiosis (also see Supplementary Table S8).

Despite their great importance during AM symbiosis, the effectors present in the secretomes of AM fungi have not been widely explored yet. Until now, only one *R. irregularis* effector SP7 has been characterized in detail (Kloppholz et al., 2011) and unfortunately, no significant homologs were found in the *G. rosea* secretome (the best hit with an *e*-value of 3E^-8^). The specificity of SP7 to *R. irregularis* agrees with the presence of many *G. rosea* specific SPs which don’t have homologs in *R. irregularis*, and indicates that each AM fungus, like plant pathogens, may also employ a specific symbiosis effector “toolkit” to manage its interactions with their host plants.

As a given AM fungus can colonize a wide range of plant species and a given plant species can be also colonized by many different AM fungi, AM symbiosis was generally assumed to be non-specific (Smith and Read, 1997). However, recent studies present evidence for the specificity or preference for certain AM fungus and plant combinations (Sanders, 2003; Croll et al., 2008). Moreover, some AM fungi can form various interaction structures, i.e., intraradical hyphal coils or arbuscules, in a given host plant (Dickson et al., 2007). Interestingly, these fungal morphological variations were observed in our samples. However, the determinants of these morphological variations and their incidence on AM symbiotic functionality are still unknown. As SPs could potentially interact with many pathways of the host, we can’t exclude the intriguing possibility that the SPs specific to certain AM fungal species determine the specificity and the differences in their abilities to form various types of AM.

### Transporter Annotation

**FIGURE 3:**
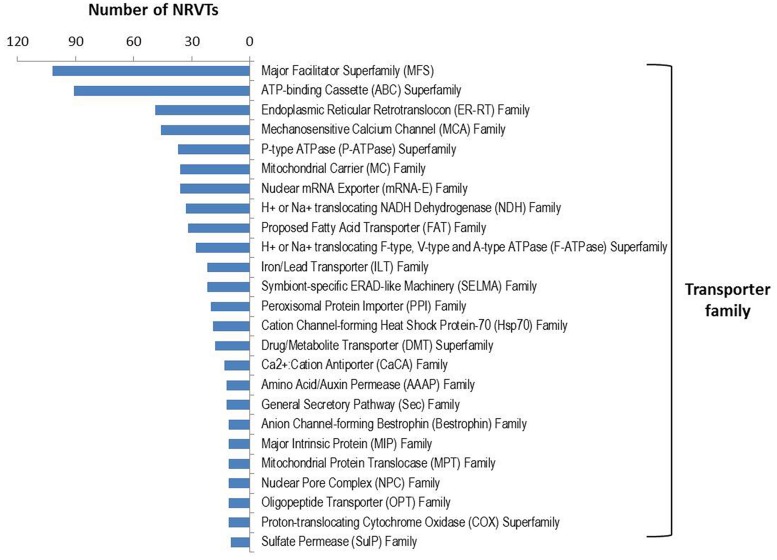
**Transporter family distribution in GiroV1.** A total of 934 NRVTs were found to encode for putative transporters among 104 families or superfamilies using blastx searches against the Transporter Classification Database. Only transporter families encoded by more than 10 NRVTs are shown.

Arbuscular mycorrhizal fungi significantly enhance host plant nutrient acquisition from the surrounding soil during AM symbiosis and in exchange, they take up the plant-fixed carbohydrates (Kiers et al., 2011). This requires an efficient nutrient transport system in which the transporters play a major role. Several genes involved in nutrient or water transport have been isolated recently in AM fungi, such as the phosphate transporters (Harrison and van Buuren, 1995; Maldonado-Mendoza et al., 2001; Benedetto et al., 2005), nitrate and ammonium transporters (López-Pedrosa et al., 2006; Tian et al., 2010; Pérez-Tienda et al., 2011; Ellerbeck et al., 2013), monosaccharide transporter (Schüssler et al., 2006; Helber et al., 2011), and aquaporins (Aroca et al., 2009; Li et al., 2013). In the gene repertoire of *G. rosea*, we found 934 NRVTs potentially coding for transporter proteins (Supplementary Table S9). These proteins belong to 104 transporter families or superfamilies in which the major facilitator superfamily (MFS) and the ATP-binding Cassette (ABC) superfamily were the most abundant transporter families (**Figure 3**). MFS transporters catalyze solute uniport, solute:cation (H^+^ or Na^+^) symport and/or solute:H^+^ or solute:solute antiport (Pao et al., 1998). The MFS transporters found in *G. rosea* are mainly putative phosphate transporters, nitrate transporters, MFS multidrug transporters, monosaccharide transporters and nicotinic acid transporters. ABC transporters utilize the energy of adenosine triphosphate (ATP) binding and hydrolysis to carry out the translocation of various substrates across membranes (Rees et al., 2009). The ABC superfamily transporters identified in *G. rosea* mainly include ABC multidrug transporters, multidrug resistance-associated proteins, MRP-like ABC transporters and Oligomycin resistance ATP-dependent permeases. In addition, several water channels or aquaporins [belonging to the major intrinsic proteins (MIPs) family] and proteins transporting ammonium, amino acids, oligopeptides, sulfate, fatty acids, and metals like zinc, iron, and magnesium were also present in this fungus. Importantly, all transporter genes isolated from other AM fungi (belonging to the orders of Glomerales and Paraglomerales) were clearly present (tblastn, *e*-value < 1E^-50^) in the *G. rosea* tranporter gene pool (Supplementary Table S9), indicating the conserved role of these transporter genes in all AM fungi and also the efficiency of our strategy for transporter annotation. Apart from these well-described genes, the current annotation also includes genes transporting other substrates like heavy metals and drugs, which may point to other potential yet unrevealed functions of AM fungi. These newly annotated genes represent valuable sequence data for further functional validation.

### Transcription Factor Gene Annotation

Transcription factors (TFs) orchestrate gene expression regulation of a cell and the repertoire of TFs determines the life and functionality of the cell (Shelest, 2008). To better understand their regulatory mechanisms, it is essential to know the repertoire of TFs in a species. To annotate the TF genes of *G. rosea*, we screened all NRVTs for the 37 PFAM domains (Todd et al., 2014) or 83 InterPro domains (Park et al., 2008), which are typically harbored in fungal TFs, and obtained 559 potential TF genes (Supplementary Table S10). Further homology searches against the Swissprot and Refseq protein databases confirmed that most are TFs or transcriptional regulator genes. Importantly, we identified a significant homolog (contig_342) in this TF repertoire of the only validated AM fungal TF gene *GintSTE*. It has been shown that GintSTE plays a key role in host infection during the early steps of AM symbiosis (Tollot et al., 2009). The finding of more than a 100 HMG-box TFs in *G. rosea* is in line with the recent identification of a large family of HMG-box TFs in *R. irregularis* and *G. margarita* (Tisserant et al., 2013; Salvioli et al., 2016). As in the Ascomycota and Basidiomycota fungi, *G. rosea* has a large gene family that contains the fungus-specific TF domain PFAM00172. Some members of this family have been reported to be involved in a variety of cell processes such as sugar and amino acid metabolism, respiration, vitamin synthesis, cell cycle, chromatin remodeling, nitrogen utilization, drug resistance, and stress responses (Shelest, 2008). Regarding the great importance and rare characterization of AM fungal TF genes, the annotation here represents the first step toward understanding regulatory mechanisms in *G. rosea*.

### Meiosis-Related Gene Identification

As no sexual stage has been observed, AM fungi have long been considered as clonal organisms, although some recombination events were reported (Croll and Sanders, 2009). However, this notion was challenged by the recent finding that most meiosis required genes are present in several *Glomus* species (Halary et al., 2011). Moreover, the identification of genes containing a mating-type high mobility group domain (MATA_HMG) and recombinations in divergent strains of *R. irregularis* strengthened the argument that AM fungi might have a cryptic sex (Tisserant et al., 2012, 2013; Riley et al., 2014). The finding of a large family of HMG-box TFs in *G. margarita* also supports this speculation (Salvioli et al., 2016). To better explore the sex potential of *G. rosea*, we searched for meiosis related genes in its gene repertoire, in addition to the identified HMG-box TFs. Our analysis identified 48 meiosis-related genes including the four meiosis-specific genes (*Mnd1, Hop2, Dmc1* and *Msh4*) reported to function exclusively in the meiosis process (Supplementary Table S11). Most of the genes found in the *Glomus* species and *R. irregularis* (Halary et al., 2011; Tisserant et al., 2013), were also present in GiroV1, indicating they could be well-conserved in the whole Glomeromycota phylum even though their exact function is not known. The lack of homologs of the other three meiotic specific genes *Rec8, Spo11*, and *Msh5* in GiroV1 could be due to the incompleteness of this assembly or to the low transcript abundance of these genes under our sequencing conditions. Although, the presence of meiosis-related genes and HMG_box genes in *G. rosea* and other AM fungi suggests the very existence of meiotic events in *G. rosea*, it doesn’t necessarily mean these genes are truly used in a typical sexual process, as they could also function in other processes (Riley and Corradi, 2013; Riley et al., 2014). As an illustration, it was recently showed in the asexual soil fungus *Fusarium oxysporum* that the sex pheromone machinery-involved STE and MAPK genes, also largely represented in our GiroV1 assembly, can also be used in the host signal perception (Turrà et al., 2015). Therefore, further functional validation of these genes and the detection of ultrastructural or genomic sexual events are urgently needed to verify the sexual ability of AM fungi (Corradi and Lildhar, 2012; Riley and Corradi, 2013; Riley et al., 2014).

### NRVTs of *G. rosea* Overexpressed In planta

Although, our knowledge on regulatory and physiological mechanisms that underlie the functionality of AM symbiosis are still patchy (Gutjahr and Parniske, 2013), it was repeatedly described that *in planta* mycelium of AM fungi have contrasted physiology compared to non-symbiotic mycelium (Benedetto et al., 2005; Wewer et al., 2014). In addition to the description of the gene repertoire of *G. rosea*, we searched for *G. rosea* genes that are specifically or over-expressed in the host. Mapping RNAseq libraries from germinating spores and symbiotic tissues led to identify 1534 genes that are significantly and highly overexpressed *in planta* (fold change > 5; FDR < 0.05, Supplementary Table S12). In this list, 360 NRVTs show a GO annotation with 61 different GO biological processes among which the most represented are oxidation–reduction process (98), protein phosphorylation (45), metabolic process (43), transmembrane transport (35), proteolysis (35), and carbohydrate metabolic process (11). As trophic exchanges are of major importance in AM symbiosis, transmembrane transporters are main markers of symbiotic functionality. A set of seven ABC transporters overexpressed was identified, among which two are quite specifically expressed *in planta* (contig_19210, contig_10203). These NRVT code for proteins whose closest homologs in *R. irregularis* are ABC-B2 transporters as recently defined by Kovalchuk et al. (2015). The role of ABC-B2 transporters is still undefined, but their overexpression both by endo- (this study) and ectomycorrhizae underline their importance in symbiotic life style. Several transporters of organic compounds are overexpressed *in planta*, like MFS glucose transporter (contig_30855), oligopeptide transporter (contig_18574). Lipid metabolism is a key metabolism for AM fungi that are oleaginous organisms. As an illustration of the highly intense fungal physiology in the symbiotic stage compared to germination spores, several NRVTs coding for lipid catabolic genes are specifically or highly expressed *in planta* as the contig_72125 (putative acyl-coA dehydrogenase, the first enzyme involved in β-oxidation of lipids), contig_57973 and contig_60441 (putative triglyceride lipase). Interestingly, one major plant gene involved in triglycerides and supposedly in suberine formation is highly overexpressed and essential for symbiosis establishment (ram2, *Glycerol-3-Phosphate Acyl Transferase*; Wang et al., 2012). It is tempting to speculate that contig_57973 and contig_60441 could be involved in feeding the fungus from plant lipids. To sustain the activation of primary metabolism, enzymatic cofactors are essential: as an illustration two NRVTs (contig_12032 and contig_25127) involved in the biosynthesis of vitamin riboflavin and pantothenate were found upregulated. As a marker of active fungal growth *in planta*, NRVTs coding for chitin synthase I (contig_32940) and chitin deacetylase (contig_51700) are overexpressed: they would contribute to meet the large demand of cell wall material for rapid proliferation of intradical hyphae and arbuscules inside root. Besides, a copper/zinc superoxide dismutase (Cu/Zn SOD) gene (contig_84388) was also found to be specifically induced in the symbiotic condition, as it has been shown that the Cu/Zn SOD gene of *G. margarita* could protect the fungus, as a reactive oxygen species-inactivating system, against localized host defense responses raised in arbuscule-containing cells (Lanfranco et al., 2005). Lastly, the presence of 13 signaling-related (especially the two-component signal transduction system) NRVTs suggests that these signaling processes play an important role during the development of symbiosis.

Among the 1534 NRVTs significantly overexpressed *in planta*, 425 were identified as specifically expressed in symbiotic tissues. Quite a half of these NRVT are specific to *G. rosea*: 222 have a putative homologous sequence in *G. margarita* gene repertoire, 105 with *R. irregularis* gene repertoire (Gloin1) and only 47 have a Refseq annotation, among which 23 are hypothetical proteins without proposed function. This large proportion of specific symbiotic NRVTs that do not show any similarity to known genes, even to the ones of the closest sequenced *G. margarita*, suggests that each AM fungal species developed lineage-specific strategy to interact with their hosts.

### Phylogenomic Analysis

Previous rDNA-phylogeny suggested placement of Glomeromycotan fungi as a sister phylum to the Dikarya (i.e., the phyla of Basidiomycota and Ascomycota; Schüβler et al., 2001). This placement has been challenged recently by several studies using nuclear or mitochontrial protein-encoding genes (Corradi and Sanders, 2006; Liu et al., 2006, 2009; Lee and Young, 2009; Nadimi et al., 2012; Lin et al., 2014), in which AM fungi usually clustered with those belonging to the Mucoromycotina. It should be noted that Mucoromycotina fungi, formerly classified as zygomycota also have coenocytic hyphae, similar to AM fungi.

In addition, recent findings that the endobacteria harbored in mucoromycotina fungi were clustered with those found in AM fungi (Desirò et al., 2015), together with the evidence that both AM fungi and Mucoromycotina fungi co-existed in the early plants (Bidartondo et al., 2011; Field et al., 2015; Rimington et al., 2015), all suggest the close relatedness of Glomeromycota with Mucoromycotina.

To reveal the relationship between AM fungi and other basal fungi clades, we first assessed the similarity of *G. rosea* genes to those of other fungi in Mucoromycotina, Basidiomycota, and Ascomycota. The results clearly showed that AM fungal genes are more similar to those of Mucoromycotina fungi, than to Basidiomycota and Ascomycota (Supplementary Table S13 and Supplementary Figure S4), thereby suggesting the close relationship between the Glomeromycota and Mucoromycotina clades.

**FIGURE 4:**
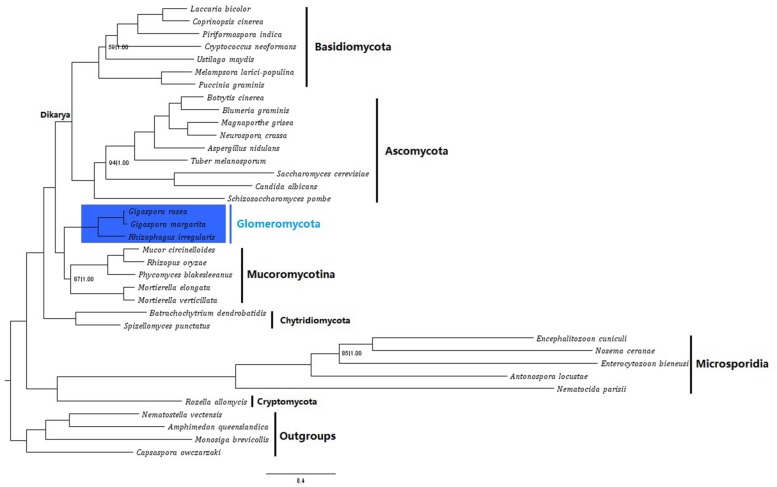
**Phylogenetic placement of *G. rosea* within fungi.** Phylogenetic trees were reconstructed on the Gblocks refined supermatrix of 118 orthologous genes, using both Bayesian and Maximum-likelihood (ML) algorithms. Only the Bayesian tree is displayed, as trees inferred by both methods were nearly identical. Statistical support values: Bootstraps (BS) followed by Bayesian posterior probabilities (BPPs) were computed for each node and only the nodes with either BS < 100 or BPP < 1.00 are indicated. The scale bar represents the number of amino acid substitutions per site.

To better resolve the relationship between AM fungi and other fungal clades, we then reconstructed a phylogenetic tree of fungi from nearly all basal lineages (including Glomeromycota, Mucoromycotina, Basidiomycota, Ascomycota, Chytridiomycota, Cryptomycota, and Microsporidia) based on a supermatrix of 118 orthologous genes (35313 amino acid sites), using both Bayesian and ML algorithms. These trees clearly showed that the AM fungi including *G. rosea* and *R. irregularis* are closely related to the subphylum Mucoromycotina, rather than to the dikarya of Basidiomycota or Ascomycota (**Figure 4**). This is in general consistent with the recent phylogenies reported in other studies. However, the phylogenetic analyses with mitochondrial genes only associated *R. irregularis* and *G. rosea* with the order Mortierellales (Lee and Young, 2009; Nadimi et al., 2012) and this was also observed in another analysis of nuclear genes (Liu et al., 2009). And, the phylogenies which clustered Glomeromycota with the order Mucorales usually didn’t include other orders (like Mortierellales and Endogonales) of Mucoromycotina, thereby the relationship between AM fungi and other Mucoromycotina orders like Mortierellales and Endogonales has still not been resolved (Corradi and Sanders, 2006; Halary et al., 2011; Lin et al., 2014; Salvioli et al., 2016). Given that the Endogonales are more closely related to the Mucorales (James et al., 2006; Bidartondo et al., 2011), our phylogenomic analysis of multiple species from nearly all basal fungal lineages, even without the data of Endogonales (no genome available yet), reveals that Glomeromycotan fungi form a sister clade to the Mucoromycotina fungi at the subphylum level, not only to the order of Mucorales or Mortierellales.

## Conclusion

Despite the great importance in ecology and potential in sustainable agriculture, progress in decoding the molecular basis of AM symbiosis from the fungi side is largely hindered by the complexity of their biology and genetics. The release of the first AM fungal genome and transcriptome of *R. irregularis* has started to shed light on the obligate biotrophy and specific genetic questions. However, with the findings from only one species, it is still difficult to assert if these features identified in *R. irregularis* represent a generality in the whole AM fungal group, considering the phylogenetic diversity in Glomeromycota. In addition to the recently published *G. margarita* transcriptome, the gene repertoire survey of *G. rosea* DAOM 194757 here provides complementary data to check the conservation of these features.

Since the Glomerales and Diversisporales might have been separated by millions of years of evolution (Dotzler et al., 2006), the identification of many lineage-specific contigs and several KOG protein families of different sizes in *G. rosae-G. margarita* and *R. irregularis* is not unexpected. But at the same time, there is a high similarity of MGCC profiles and a high conservation of certain gene categories. These genomic features shared across different orders in Glomeromycota may mirror the conserved life style of AM fungi in general, such as the mutualistic obligate biotrophy and the as yet undescribed sexual mechanism. Compared to pathogenic fungi, AM fungi are characterized by a dual ability both to intimately interact with the host and to transfer nutrients from the surrounding environment to their host. Additionally, our phylogenomic analysis of basal fungal clades clearly revealed the close relationship between the Glomeromycotan and Mucoromycotina fungi, thus validating the recent suggested placement of AM fungi in a clade sister to the Mucoromycotina.

The release of this new gene repertoire not only enables the global gene repertoire comparison with fungi from other clades of Glomeromycota, but also provides a large number of gene candidates for further functional characterization.

## Author Contributions

CR, GB, and BZ conceived this research; NT and SR prepared the biological material for sequencing; NT and HSC performed the data analysis; NT and CR wrote the manuscript, and CR, GB, and BZ revised the manuscript.

## Conflict of Interest Statement

The authors declare that the research was conducted in the absence of any commercial or financial relationships that could be construed as a potential conflict of interest.

## Footnotes

1 http://get.genotoul.fr/

2 www.clcsupport.com/clcgenomicsworkbench/current/index.php?manual=Introduction_CLC_Genomics_Workbench.html

3 www.cbs.dtu.dk/services/SignalP/

4 www.cbs.dtu.dk/services/TMHMM/

5 www.tcdb.org/

6 www.broadinstitute.org/

7 www.yeastgenome.org/

8 http://www.atgc-montpellier.fr/phyml/
