# Supplementary material for: A Survey of the Gene Repertoire of Gigaspora rosea Unravels Conserved Features among Glomeromycota for Obligate Biotrophy
Source: Front Microbiol. 2016 Mar 1;7:233. doi: 10.3389/fmicb.2016.00233 (PMC4771724; doi:10.3389/fmicb.2016.00233)
Supplement: Supplementary file 1 [file Data_Sheet_1.ZIP › Figure S1. Strategy for the cleaning of symbiotic reads and GC content distribution in the cleaned symbiotic reads.pdf]

(A)

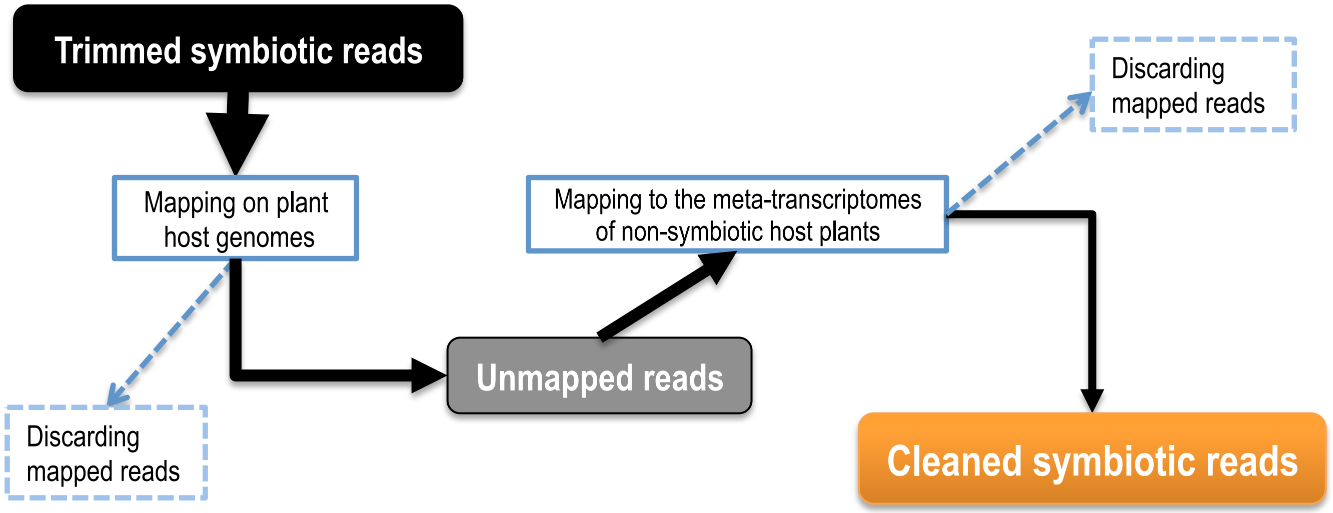

(B)

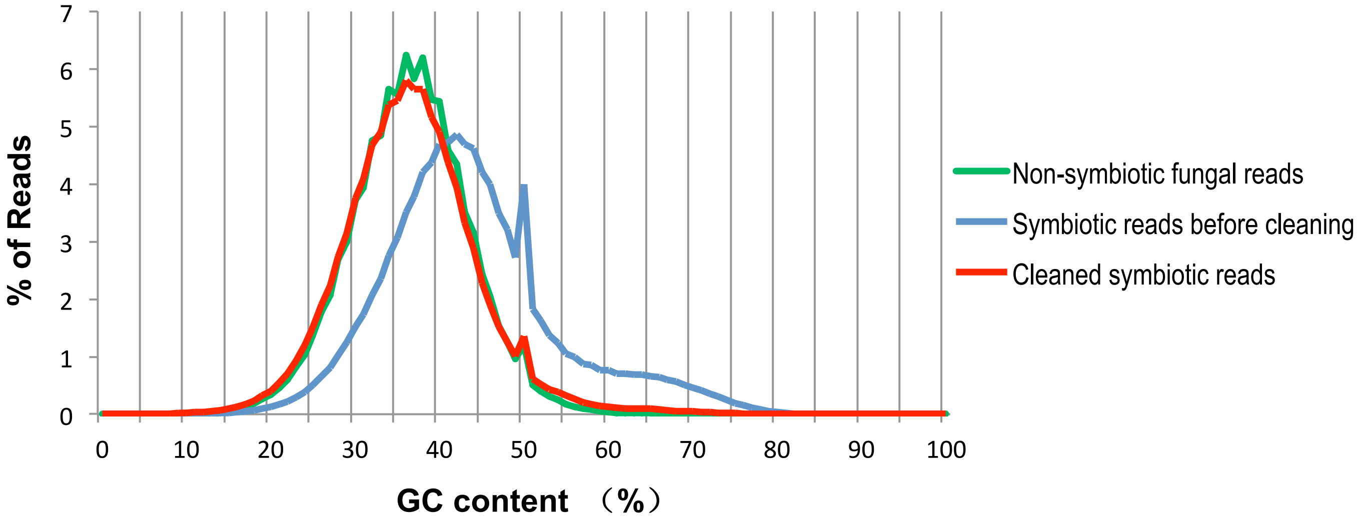

Figure S1. Strategy for the cleaning of symbiotic reads (A) and GC content distribution in the cleaned symbiotic reads (B).
(A)strategy for the cleaning of symbiotic reads: the CLC trimmed in planta reads were firstly mapped onto the genomes of host plants (medicago and brachypodium); After discarding the mapped reads the remaining reads were further mapped onto the meta-transcriptomes of non-mycorrhized control host roots in order to remove the other potential eukaryotic contaminant reads which might be introduced in the greenhouse condition. (B) The symbiotic reads after cleaning displays a GC% distribution (red) that is very similar to that of the non-symbiotic fungal reads (green), as compared with the in planta reads before the cleaning (blue).
