# Supplementary material for: A Survey of the Gene Repertoire of Gigaspora rosea Unravels Conserved Features among Glomeromycota for Obligate Biotrophy
Source: Front Microbiol. 2016 Mar 1;7:233. doi: 10.3389/fmicb.2016.00233 (PMC4771724; doi:10.3389/fmicb.2016.00233)
Supplement: Supplementary file 1 [file Data_Sheet_1.ZIP › Figure S2. GO category distribution in GiroV1.pdf]

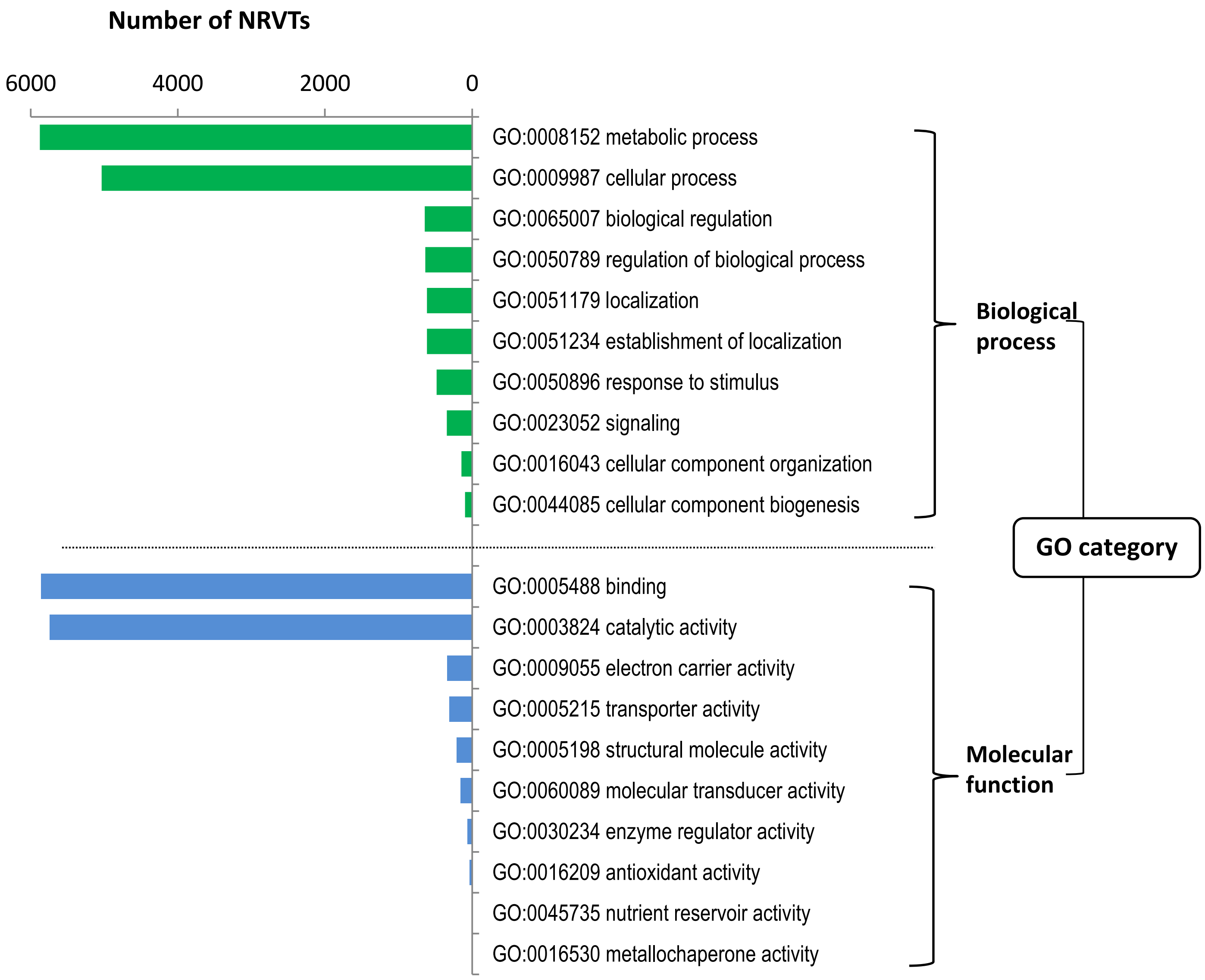

Figure S2. GO category distribution in GiroV1

The number of NRVTs assigned into each Gene Ontology biological process (green) and molecular function (blue) category, at the level two, was calculated as the percentage of the total assigned NRVTs. The top presented 10 biological processes and all molecular functions were showed.
