# Supplementary material for: A Survey of the Gene Repertoire of Gigaspora rosea Unravels Conserved Features among Glomeromycota for Obligate Biotrophy
Source: Front Microbiol. 2016 Mar 1;7:233. doi: 10.3389/fmicb.2016.00233 (PMC4771724; doi:10.3389/fmicb.2016.00233)
Supplement: Supplementary file 1 [file Data_Sheet_1.ZIP › Figure S3. InterPro domains distribution in the gene repertoire of G. rosea.pdf]

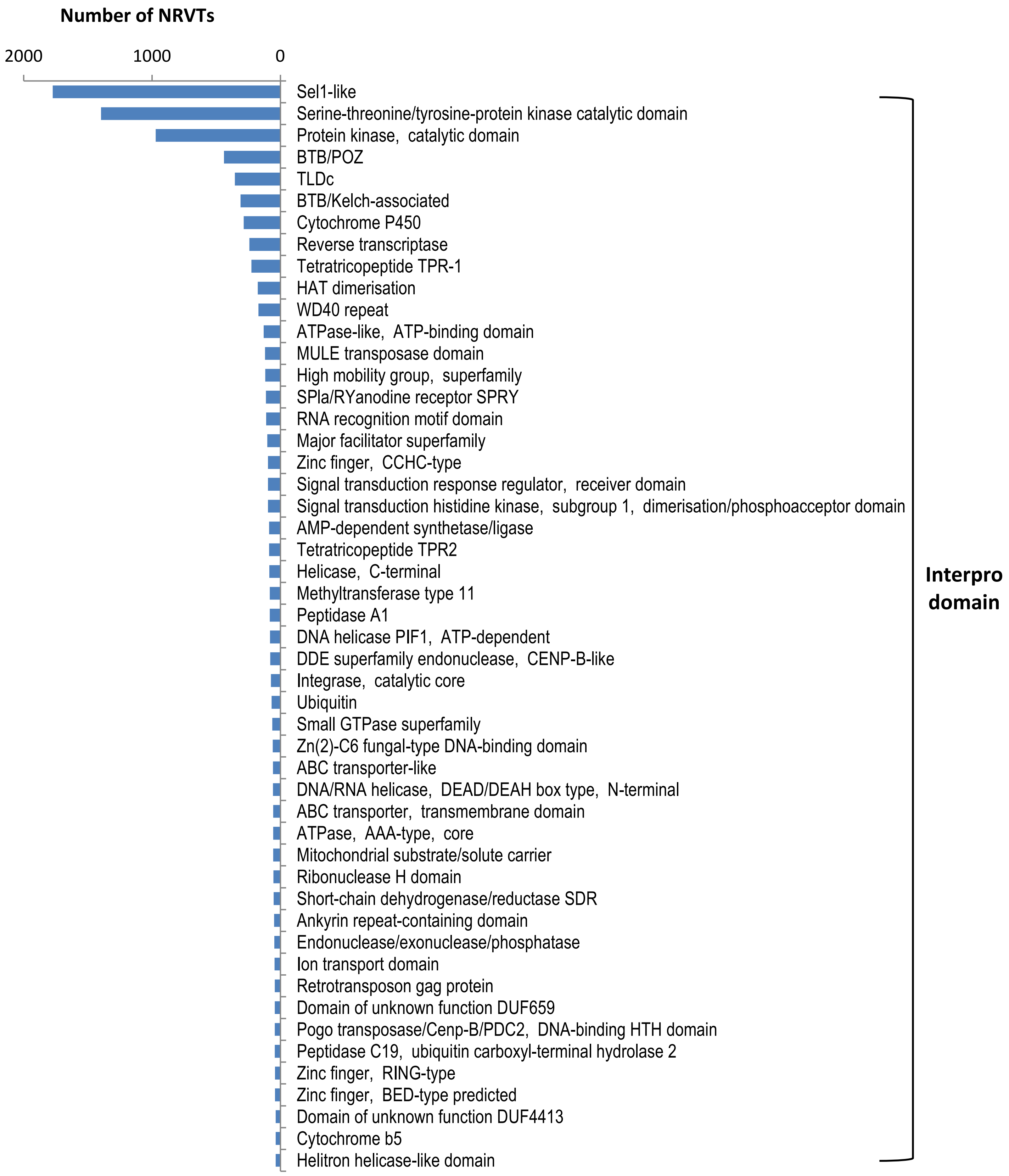

Figure S3. InterPro domains distribution in the gene repertoire of *G. rosea*

A total of 3076 interpro domains were found in 14413 G.rosea NRVTs using InterProscan program. Only the top 50 presented domains are showed.
