# Supplementary material for: A Survey of the Gene Repertoire of Gigaspora rosea Unravels Conserved Features among Glomeromycota for Obligate Biotrophy
Source: Front Microbiol. 2016 Mar 1;7:233. doi: 10.3389/fmicb.2016.00233 (PMC4771724; doi:10.3389/fmicb.2016.00233)
Supplement: Supplementary file 1 [file Data_Sheet_1.ZIP › Figure S4. Similarity of G. rosea NRVTs to the homologous genes of other representative fungi.pdf]

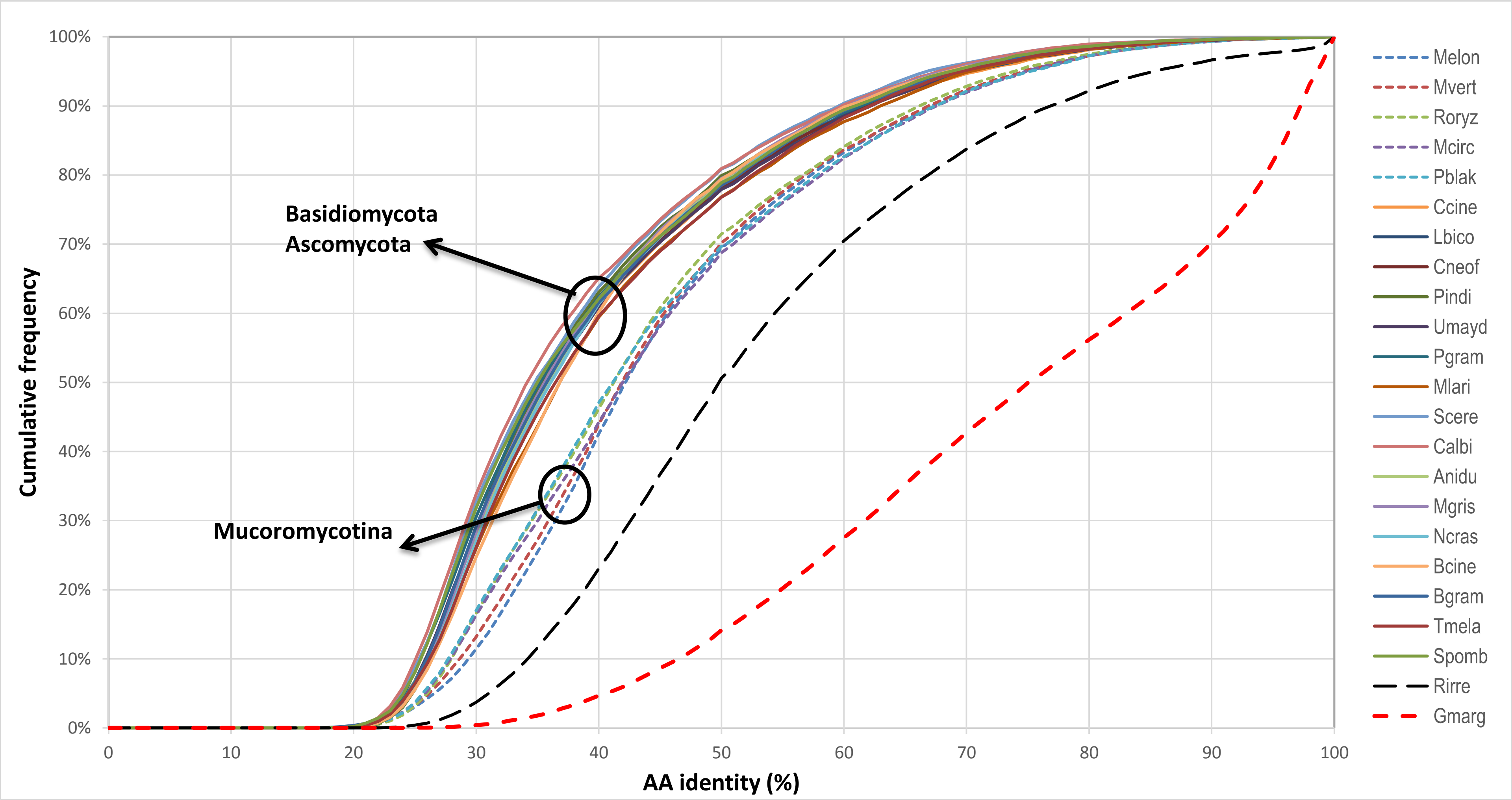


**Figure S4. Similarity of *G. rosea* NRVTs to the homologous genes of other representative fungi**

Cumulative frequencies of AA identity across each set of homologs. Homologs were obtained by blastx *G.rosea* NRVTs onto the target genomes. Percentages of *G.rosea* NRVTs showing different level of AA identities were accumulated (from the lowest to highest AA identity). Fungal species abbr. Rirre for *Rhizophagus irregularis*, Gmarg for *Gigaspora margarita*, Melon for *Mortierella elongata*, Mvert for *Mortierella verticillata*, Roryz for *Rhizopus oryzae*, Mcirc for *Mucor circinelloides*, Pblak for *Phycomyces blakesleeanus*, Ccine for *Coprinopsis cinerea*, Lbico for *Laccaria bicolor*, Cneof for *Cryptococcus neoformans,* Pindi for *Piriformospora indica*, Umayd for *Ustilago maydis*, Pgram for *Puccinia graminis*, Mlari for *Melampsora larici-populina,* Scere for *Saccharomyces cerevisiae*, Calbi for *Candida albicans*, Anidu for *Aspergillus nidulans*, Mgris for *Magnaporthe grisea*, Ncras for *Neurospora crassa,* Bcine for *Botrytis cinerea*, Bgram for *Blumeria graminis*, Tmela for *Tuber melanosporum* and Spomb for *Schizosaccharomyces pombe.*
